# Development and Validation of a Decision Tree Analysis Model for Predicting Home Discharge in a Convalescent Ward: A Single Institution Study

**DOI:** 10.1298/ptr.E10267

**Published:** 2024-01-19

**Authors:** Dai NAKAIZUMI, Shingo MIYATA, Keita UCHIYAMA, Ikki TAKAHASHI

**Affiliations:** ^1^Department of Rehabilitation, Japanese Red Cross Kanazawa Hospital, Japan; ^2^Department of Physical Therapy, Graduate Course of Rehabilitation Science, School of Health Sciences, College of Medical, Pharmaceutical, and Health Sciences, Kanazawa University, Japan; ^3^Department of Rehabilitation, Suzuki Clinic Orthopaedics River City, Japan

**Keywords:** Decision trees, Patient discharge, Clinical decision-making, Activities of daily living

## Abstract

Objectives: Accurately predicting the likelihood of inpatients’ home discharge in a convalescent ward is crucial for assisting patients and families in decision-making. While logistic regression analysis has been commonly used, its complexity limits practicality in clinical settings. We focused on decision tree analysis, which is visually straightforward. This study aimed to develop and validate the accuracy of a prediction model for home discharge for inpatients in a convalescent ward using a decision tree analysis. Methods: The cohort consisted of 651 patients admitted to our convalescent ward from 2018 to 2020. We collected data from medical records, including disease classification, sex, age, duration of acute hospitalization, discharge destination (home or nonhome), and Functional Independence Measure (FIM) subitems at admission. We divided the cohort data into training and validation sets and developed a prediction model using decision tree analysis with discharge destination as the target and other variables as predictors. The model’s accuracy was validated using the validation data set. Results: The decision tree model identified FIM grooming as the first single discriminator of home discharge, diverging at four points and identifying subsequent branching for the duration of acute hospitalization. The model’s accuracy was 86.7%, with a sensitivity of 0.96, specificity of 0.52, positive predictive accuracy of 0.88, and negative predictive accuracy of 0.80. The area under the receiver operating characteristic curve was 0.75. Conclusion: The predictive model demonstrated more than moderate predictive accuracy, suggesting its utility in clinical practice. Grooming emerged as a variable with the highest explanatory power for determining home discharge.

## Introduction

In Japan, the “Kaifukuki Rehabilitation Ward,” a convalescent ward where intensive and comprehensive inpatient rehabilitation is carried out, was established in 2000. This ward is designed to enhance the activities of daily living in alignment with the social background of target patients and their families and to facilitate their return home and reintegration into society^1)^. The accurate prediction of the likelihood of home discharge for inpatients in these convalescent wards is crucial, as it aids the decision-making processes for both patients and their families and promotes early discharge support. Predicting the likelihood of home discharge using data from the time of admission would be beneficial for facilitating discharge from the early stage of admission to convalescent wards.

The predictions concerning the feasibility of home discharge depend on a multitude of factors, and as a result, many preceding studies have employed logistic regression analyses^2–5)^. While this is an effective method for evaluating the influence of individual factors, it might be challenging to apply clinically due to the complexity of the predictive equation. Furthermore, although it allows for the identification of the odds ratios of each explanatory variable, it falls short of making the interrelationships among variables easily discernible.

To compensate for these shortcomings, we focused on the decision tree method, a machine learning technique. Machine learning algorithms have been widely used in recent years to predict clinical outcomes^6,7)^. The decision tree analysis method, first reported by Briunam^8)^, is characterized by its capacity to present the results of the analysis using a tree structure, which is visually intuitive. This method provides a threshold value for explanatory variables to divide data into subsets with similar values of the objective variable in a stepwise manner. As all explanatory variables are stratified in order of relevance, the interrelationships among factors become clear, thus simplifying their clinical application.

In previous studies predicting discharge destinations, the accuracy of the prediction model was often validated within the same population used to develop the model^9–13)^. Therefore, even if the predictive accuracy is high, there is a high likelihood of overfitting, a condition where the model excessively adapts to the population used for its creation. Overfitting occurs when a model learns noise or random patterns from the training data, leading to reduced predictive accuracy on new data. Using an overfitted model in a clinical setting increases the likelihood of making incorrect predictions. Therefore, it is essential to verify the generalization capability of predictive models. In this study, we employed k-fold cross-validation to develop and validate the accuracy of the prediction model.

This study aimed to develop a prediction model for home discharge for inpatients admitted to a convalescent ward using a decision tree analysis to test its accuracy across different populations.

## Methods

### Cohort composition and exclusions

The study cohort consisted of all 651 patients who were admitted to a single convalescent ward between the fiscal years of 2018 and 2020. Patients who were discharged due to death, transferred due to an acute exacerbation, or transferred to an acute care ward were excluded from the study.

### Data collection and measures

We retrospectively collected data from the medical records of the participants, including disease classification (cerebrovascular disease, musculoskeletal disease, disuse syndrome), sex assigned at birth (based on visible external anatomy), age, duration of acute hospitalization, discharge destination (home, nonhome), and the subitems of the Functional Independence Measure (FIM) at the time of admission to the convalescent rehabilitation ward. We employed the Japanese version of FIM version 3.0^14,15)^, which incorporates culturally relevant modifications for some items^16,17)^. FIM is a tool that quantifies the severity of disability in rehabilitation patients, consisting of 18 items: 13 on motor functions and 5 on cognitive functions^18)^. The motor subscale of the FIM includes the following 13 items: eating, grooming, bathing, dressing the upper body, dressing the lower body, toileting, bladder management, bowel management, transfer to bed/chair/wheelchair, transfer to toilet, transfer to tub/shower, locomotion (walk/wheelchair), and stairs. The cognitive subscale of the FIM includes the following 5 items: comprehension, expression, social interaction, problem solving, and memory. The FIM item scores are systemically graded on a seven-point scale (1, total assistance; 7, complete independence). The FIM assessment upon admission to the ward was collaboratively conducted by the physical therapist assigned to each patient, the occupational therapist, the speech therapist, and the ward nurse. No specific examiner was chosen as the evaluation was performed within the routine operations. In this retrospective study, all necessary data were comprehensively recorded, and as a result, there were no missing values in the dataset. Therefore, there was no need for handling missing data using methods such as complete-case analysis, single imputation, or multiple imputation.

### Data allocation and comparative analysis

Seventy percent of the study subjects were randomly allocated to the training data, and the remaining 30% to the validation data. We conducted an intergroup comparison of each factor between patients in the training data and those in the validation data. Fisher’s exact probability test was used for disease classification, the chi-squared test was used for sex, and Mann–Whitney’s U test was applied to age, duration of acute hospitalization, and subitems of the FIM.

### Development and evaluation of the prediction model

A predictive model was developed using the training data through a decision tree analysis (classification and regression tree: CART). Decision tree analysis is a graphical method for classifying or predicting data, which iteratively asks questions based on input data characteristics, forming a “tree”-shaped model. Within decision tree analysis, the CART method is the primary method for generating models^8,18,19)^. The CART method uses a binary tree structure that divides the data into two subsets based on specific questions. Each question relates to an attribute (e.g., disease classification, gender, and age) and directs the pathway to the next branch based on the answer. Upon reaching the lowest node (leaf), it signifies the result of data classification. In generating the CART method, the Gini coefficient was employed as the node-splitting criterion. It assesses node impurity: values near 0 indicate high purity, and those close to 1 show high impurity. This study’s analysis divided data to minimize the Gini coefficient. The prediction model used discharge destination as the objective variable and disease category, sex, age, length of acute hospitalization, and the FIM subitems at admission to the recovery unit as explanatory variables. In decision tree analysis, the complexity parameter (CP) value controls tree growth. A high cp value yields a simpler tree, risking underlearning as it may not fully capture data patterns. Conversely, a low cp value can lead to overlearning. To optimize the cp value, 10-fold cross-validation was employed on the training data. This involved dividing it into 10 subsets, training on 9, and evaluating on the remaining one, repeated 10 times. The optimal cp value was then applied to construct the final model.

The accuracy of the prediction model was evaluated using the validation data. According to the created prediction model, we predicted the discharge destination and calculated the classification accuracy, sensitivity, specificity, positive predictive value, negative predictive value, and area under the receiver operating characteristic curve (AUC–ROC) for evaluation.

### Statistical analysis

The statistical analysis was performed with R statistical software version 4.2.2 (R Foundation for Statistical Computing, Vienna, Austria), with the significance level set at 0.01.

### Ethics approval

This retrospective study was conducted with approval from the Japanese Red Cross Kanazawa Hospital’s ethics review committee (No. 543). We strictly managed privacy protection and personal information to avoid any privacy problems associated with the medical treatment.

## Results

### Cohort and dataset characteristics

**Fig. 1.**
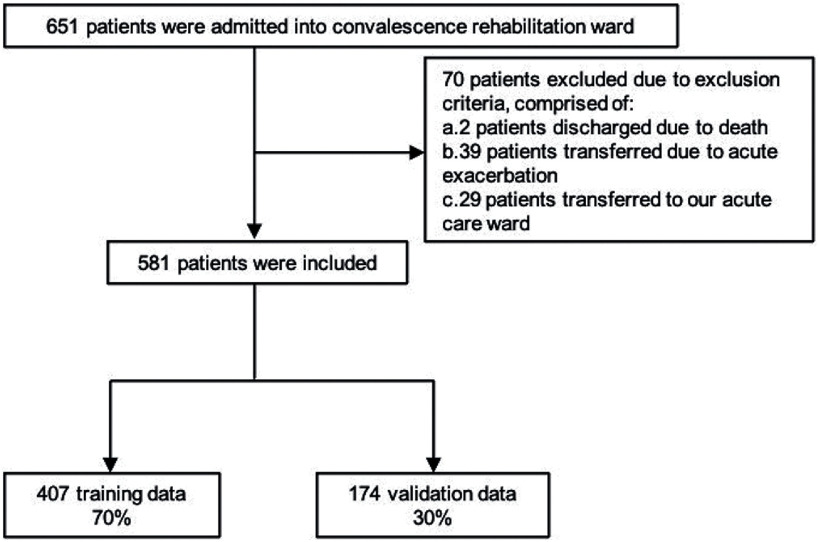
Study flowchart Decision tree algorithm analyses were conducted using 407 training data points, and accuracy was verified with 174 validation data points.

Of the 651 individuals in the cohort, 581 were included in the final analysis, while 70 were excluded due to transfers to an acute ward or death during hospitalization resulting from severe medical complications. Specifically, 2 were discharged due to death, 39 were transferred due to acute exacerbations, and 29 were transferred to our acute care ward. The training data set consisted of 407 patients, while the validation data set consisted of 174 patients (Fig. 1). The characteristics of the patients in the training and validation data sets are presented in Table 1. There were no significant differences between the training and validation data across all factors.

**Table 1.** Baseline characteristics of the analyzed subjects

Characteristics	Overall (n = 581)	Training data (n = 407)	Validation data (n = 174)	p-value
Disease classification				0.518
Motor disorder	365 (63%)	250 (61%)	115 (66%)	
Vascular brain disease	205 (35%)	148 (36%)	57 (33%)	
Disuse atrophy	11 (1.9%)	9 (2.2%)	2 (1.1%)	
Age	80 (72–87)	80 (72–87)	79 (70–87)	0.512
Sex				0.926
Female	369 (64%)	258 (63%)	111 (64%)	
Male	212 (36%)	149 (37%)	63 (36%)	
Duration of acute hospitalization	24 (15– 36)	24 (15– 35)	24 (15– 37)	0.992
Eating	6 (5–7)	6 (5–7)	7 (5–7)	0.570
Grooming	5 (4–7)	5 (4–7)	5 (4–7)	0.205
Bathing	3 (1–5)	3 (1–5)	3 (1–5)	0.790
Dressing upper body	1 (1–3)	1 (1–3)	1 (1–3)	0.682
Dressing lower body	1 (1–2)	1 (1–2)	1 (1–2)	0.651
Toileting	5 (1–6)	5 (2–6)	5 (1–6)	0.902
Bladder management	7 (1–7)	7 (1–7)	7 (1–7)	0.530
Bowel management	6 (5–7)	6 (5–7)	6 (4–7)	0.738
Transfers (bed/chair/wheelchair)	5 (3–6)	5 (3–6)	5 (3–6)	0.689
Transfers (toilet)	5 (3–6)	5 (3–6)	5 (3–6)	0.575
Transfers (bath/shower)	4 (1–5)	4 (1–5)	4 (1–5)	0.838
Locomotion (walking/wheelchair)	2 (1–5)	2 (1–5)	2 (1–5)	0.632
Stairs	1 (1–1)	1 (1–1)	1 (1–1)	0.705
Motor subtotal score	52 (33–64)	53 (33–64)	51 (34–65)	0.903
Comprehension	6 (4–7)	6 (4–7)	6 (4–7)	0.492
Expression	7 (5–7)	7 (5–7)	7 (5–7)	0.581
Social interaction	7 (7–7)	7 (7–7)	7 (7–7)	0.718
Problem solving	5 (3–6)	5 (3–6)	5 (2–6)	0.873
Memory	5 (3–7)	5 (3–7)	6 (3–7)	0.189
Cognitive subtotal score	30 (21–33)	29 (21–33)	30 (21–33)	0.580
Total FIM score	80 (58–96)	80 (58–96)	80 (57–98)	0.750
Discharge destination				0.994
Home	454 (78%)	318 (78%)	136 (78%)	
Nonhome	127 (22%)	89 (22%)	38 (22%)	

Data are presented as median (IQR).

FIM, Functional Independence Measure; IQR, interquartile range

### Decision tree for home discharge

**Fig. 2.**
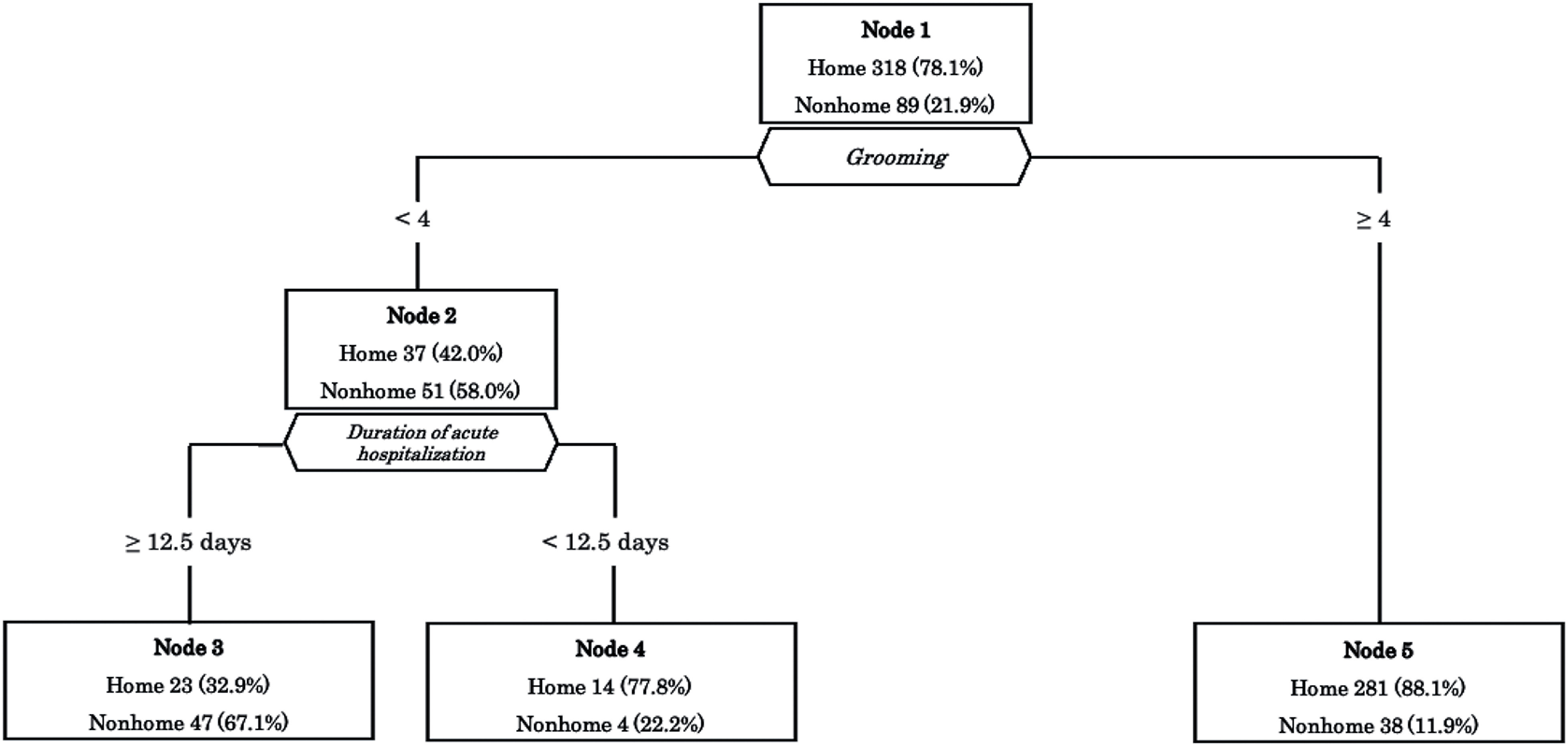
The decision tree analysis for predicting home discharge The model identified grooming in the FIM as the first single discriminating factor, with the subsequent discriminator being the duration of acute hospitalization. FIM, Functional Independence Measure

The results of the decision tree analysis are shown in Figure 2. In the decision tree analysis, the model identified grooming as the first single discriminator for home discharge, diverging based on four points. For the group with a grooming score of ≥4, discharge to home was the outcome. Conversely, for those with a grooming score of <4, the subsequent discriminator was the duration of acute hospitalization, branching at 12.5 days. This led to a predictive model in which a stay of ≥12.5 days resulted in a nonhome discharge, while a stay of <12.5 days led to a home discharge.

### Model performance metrics

**Fig. 3.**
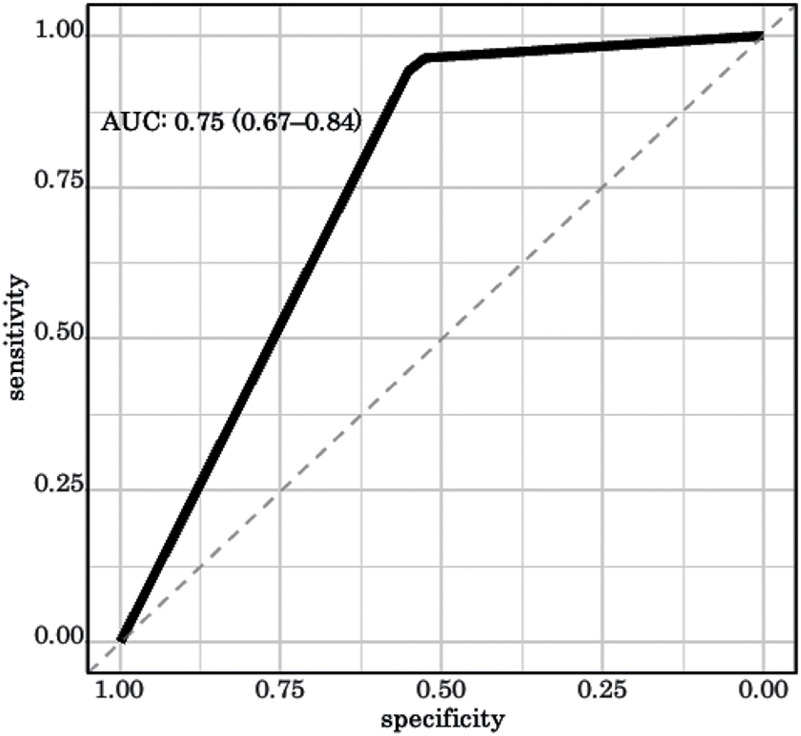
ROC curve Classification performance of home discharge using decision tree model. ROC, receiver operating characteristics; AUC–ROC: area under the receiver operating characteristic curve

The accuracy of the model with the validation data was 86.7% (95% confidence interval [CI]: 80.8%–91.4%). The sensitivity was 0.96 (95% CI: 0.92–0.98), the specificity was 0.52 (95% CI: 0.37–0.66), the positive predictive value was 0.88 (95% CI: 0.82–0.92), and the negative predictive value was 0.80 (95% CI: 0.61–0.92). The AUC–ROC was 0.75 (95% CI: 0.67–0.84) (Fig. 3).

## Discussion

This study used a decision tree analysis to create a prediction model for home discharge of patients admitted to a single convalescent rehabilitation ward, and its accuracy was validated using validation data. The predictive model created in this study showed an accuracy of 86.7%, sensitivity of 0.90, specificity of 0.52, positive predictive value of 0.88, negative predictive value of 0.80, and AUC–ROC of 0.75.

In a previous Japanese study on the likelihood of discharge to home for patients admitted to a convalescent ward using a logistic regression analysis, the accuracy was 87.8%^2)^, 88.3%^20)^, and the AUC–ROC was reported to be 0.827–0.866^12)^, 0.891^21)^. The accuracy and AUC–ROC in these previous studies were higher in comparison to the present study. However, there is a possibility of overfitting, as the data used in the logistic regression analysis and the data used to verify accuracy were identical. In previous studies that used decision tree analysis to examine home discharge of stroke patients admitted to a convalescent rehabilitation ward, the accuracy was between 78.8% and 84.8%^22,23)^, which is similar to the results of the present study. Although this study was not limited to stroke patients, the predictive accuracy was comparable to that of previous studies.

The predictive model in this study had a high sensitivity and positive predictive value. Therefore, patients who were predicted to be discharged to home by the model were more likely to be discharged to home. Conversely, the specificity was low. This suggests that the accuracy of predicting patients who will be discharged to a nonhome is low. When nonhome discharge is predicted using this model, it is necessary to consider other factors as well.

The likelihood of home discharge after rehabilitation for patients with stroke or proximal femoral fractures is reported to depend not only on the individual’s capabilities but also on social factors such as the presence and number of cohabitants and their caregiving capacity^24–27)^. Therefore, these factors must also be considered. The AUC–ROC for this prediction model was 0.81; the AUC–ROC ranges from 0.50 to 1.00 and is interpreted as “high accuracy” at 0.90 to 1.00, “moderate accuracy” at 0.70 to 0.90, and “low accuracy” at 0.50 to 0.70^28–30)^. Based on the above, the predictive model in this study had moderate forecasting performance.

In this study, regarding the predictive model, grooming was the first single discriminator of home discharge and diverged based on four points. grooming was chosen as the variable with the most explanatory power in determining the likelihood of home discharge. Jackson et al.^31)^ have shown that grooming is included as a factor in the predictive model for home discharge of stroke patients, but the threshold for this variable has not been provided. Grooming includes activities such as brushing teeth, combing hair, washing hands and face, and shaving or applying makeup. A score of 4 in grooming corresponds to a level where ≥75% of the activity is performed independently. Specifically, it is defined as a level where one can perform four out of the five constituent tasks independently, requiring assistance for the remaining one, or a level where minimal assistance is needed for all five tasks^14,15)^.

Reports suggest that the independence of grooming tasks in stroke patients is associated with factors such as seated balance, upper limb function, motivation, and cognitive function^32,33)^. The higher likelihood of home discharge with a grooming score of ≥4 could be attributed to these abilities being relatively preserved at the time of admission to the convalescent rehabilitation ward. However, a functional evaluation was not performed in this study, so the specifics could not be clarified. Additionally, the findings imply that execution functions, such as the handling of objects and understanding of operational procedures, might be associated with grooming. The results suggest that if a patient possesses the capability of performing grooming activities with light assistance at the time of convalescent ward admission, the likelihood of being discharged home is higher. Subsequent branching points, the duration of acute hospitalization, have been reported in a study as factors influencing the discharge destination^21)^. For the duration of acute hospitalization, shorter stays have been reported to increase the likelihood of home discharge. The predictive model developed in this study also incorporates this factor, indicating that a shorter duration of acute hospitalization, increases the likelihood of home discharge. These results support the findings of a previous study.

This predictive model is created with data available at the time of admission to a convalescent ward, enabling its use early after admission, such as at the initial conference. The model can help indicate the likely direction of a patient’s discharge from the ward at an early stage and assist in planning rehabilitation programs, establishing the environment, and providing care guidance to family members accordingly.

The present study was associated with some limitations. First, the explanatory variables in this study do not incorporate information about the patient’s social background or physical functional assessments into the predictive model. The decision to discharge a patient to home depends not only on the patient’s capabilities but also on their housing environment, cohabitants, and caregiving capacity. Additionally, integrating individual functional evaluation indices such as the severity of the patient’s paralysis, muscle strength, walking ability, and balance into the model could potentially improve its accuracy. Second, this study has exclusion criteria; thus, it cannot be applied to severely ill patients who experienced serious complications or death during the rehabilitation period. Third, as the study was conducted at a single institution, there are limitations to its generalizability. Despite these limitations, the predictive model created in this study, which does not categorize diseases and demonstrated moderate or higher predictive accuracy in the validation data, is believed to be a highly convenient model for clinical use.

## Conclusion

We created a predictive model for home discharge of patients admitted to a convalescent rehabilitation ward using a decision tree analysis and verified its accuracy with validation data. The model demonstrated an accuracy of 86.7%, sensitivity of 0.96, specificity of 0.52, positive predictive value of 0.88, negative predictive value of 0.80, and AUC–ROC of 0.75, indicating moderate predictive accuracy. The most explanatory variable selected for determining the possibility of home discharge was grooming performance. A subsequent factor in the predictive model was the duration of acute hospitalization. As the predictive model constructed in this study does not categorize diseases and demonstrated moderate or higher predictive accuracy in the validation data, it is therefore considered to be a very clinically convenient model.

## Conflict of Interest

The authors declare no conflicts of interest.
